# From variant of unknown significance to likely pathogenic: Characterization and pathogenicity determination of a large genomic deletion in the MLH1 gene

**DOI:** 10.1002/mgg3.2231

**Published:** 2023-06-23

**Authors:** Ahmed Bouras, Clementine Legrand, Jihen Kourda, Eric Ruano, Chloé Grand‐Masson, Cedrick Lefol, Qing Wang

**Affiliations:** ^1^ Laboratory of Constitutional Genetics for Frequent Cancer HCL‐CLB Centre Léon Bérard Lyon France; ^2^ Genetic Service, Department of Genetics and Procreation CHU Grenoble Alpes Grenoble France; ^3^ Department of Pathology CYPATH Grenoble France

**Keywords:** dMMR, large genomic rearrangement, Lynch syndrome, *MLH1*, NGS, RNA analysis

## Abstract

**Background:**

The *MLH1* gene is one of the DNA mismatch repair genes (MMR), implicated in Lynch syndrome (LS), an autosomal dominant hereditary tumor susceptibility disease. The advent of next‐generation sequencing (NGS) technologies has accelerated the diagnosis of inherited diseases and increased the percentage of diagnosis of inherited cancers. However, some complex genomic alterations require the combination of several analytical strategies to allow correct biological interpretations. Here, we describe a novel *MLH1* deletion and its pathogenicity determination in a patient suspected of LS.

**Methods:**

The index case was a French 73‐year‐old man diagnosed with colorectal cancer displaying microsatellite instability and the loss of MLH1 and PMS2 expression. NGS analysis was used as the primary method for MMR genes screening. Long‐range PCR and reverse transcriptase polymerase chain reaction (RT‐PCR) were used for breakpoints and pathogenicity determinations.

**Results:**

A large genomic deletion was detected which removed the last six nucleotides of *MLH1* exon 11 together with a large part of intron 11. It was initially considered as a variant of unknown significance (VUS). Genomic breakpoints were subsequently characterized defining the deletion as c.1033_1039‐248del. Further RNA analysis demonstrated that this variant activated a cryptic donor splice site at the 5′ of the breakpoint, leading to a premature truncated protein: p.Thr345Alafs*13.

**Conclusion:**

Our finding suggested that although NGS technologies have increased variant detection yield, combined approaches were still needed for complex variant characterization and pathogenicity assessment.

## Graphical abstract

- Combined approaches were necessary for complex variant characterization.
- Assessment of the pathogenicity of a novel genomic rearrangement of the MLH1 gene.
- Functional RNA analysis is important to define variant classification in hereditary cancer syndromes.

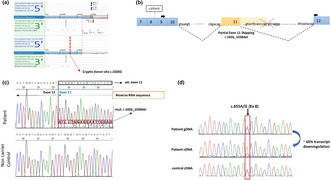

## 1 INTRODUCTION

Lynch syndrome (LS; OMIM 120435) is an autosomal dominant cancer predisposition syndrome with a prevalence of 1% to 3% in unselected colorectal cancer patients (de la Chapelle, 2005). LS is caused by germline pathogenic variants in one of the mismatch repair (MMR) genes: *MLH1, MSH2, MSH6*, and *PMS2* or 3′ deletion of the *EPCAM* gene. Tumors from LS patients show high microsatellite instability (MSI) and loss of expression of one or more MMR proteins (Boland et al., 2008). Screening for germline variants of MMR genes is systematically offered to patients either with the family history fulfilling the criteria of Amsterdam I and II or suggestive of LS with MSI phenotype and/or loss the expression of MMR protein expression in their tumors (Umar et al., 2004; Vasen et al., 1999).

Until the last decade, MMR mutations screening was mainly performed by conventional first‐generation sequencing (Sanger sequencing) complemented by other methods for CNVs detection such as Multiplex Ligation‐dependent Probe Amplification (MLPA) or Quantitative Multiplex PCR of Short fluorescent Fragment (QMPSF) (Kurzawski et al., 2006). Today, the next‐generation sequencing (NGS) has increasingly been used and become the golden standard in identification of pathogenic germline variants in hereditary cancer syndromes since it can detect not only SNVs but also CNVs. The use of gene panels has increased the diagnostic yield of hereditary cancers (Samadder et al., 2021). However, for complex genomic alterations especially those of large size of abnormalities including intronic sequences, combined approached is still necessary allowing for correct biological interpretations.

Here, we report the detection of a large deletion within *MLH1* gene in a patient suspected for LS and its pathogenicity determination.

## 2 MATERIALS AND METHODS

### 2.1 Germline variant detection

Total genomic DNA was extracted from blood sample using the automated procedure implemented on the STARlet platform (Hamilton Company, Reno, NV, USA). Next‐generation sequencing (NGS) was performed using customized Agilent XTHS panel with capture‐based target enrichment (Agilent, Santa Clara, USA). This panel included 14 genes: *MLH1* (LRG_216t1), *MSH2* (LRG_218t1), *MSH6* (LRG_219t1), *PMS2* (LRG_161t1), *POLE* (LRG_789), *POLD1* (LRG_785), *EPCAM* (LRG_215), *APC* (LRG_130), *MUTYH* (LRG_220), *STK11* (LRG_319), *BMPR1A* (LRG_298), *PTEN* (LRG_311), *CDH1* (LRG_301), and *SMAD4* (LRG_318). Sequence alignment and variant calling were carried out using an in‐house bioinformatics pipeline.

Long‐range PCR was performed using TaKaRa LA Taq (TaKaRa Bio, Osaka, Japan) and the following primers: MLH1_11F (located at the end of intron 10): GGGCTTTTTCTCCCCCTCCC and MLH1_12R (located at the inton 12): CATGAAAAGCCAAAGTTAGAAGGCAGTT at conditions: 94°C for 1 min followed by 35 cycles at 94°C for 20 s, 60°C for 30 s, 68°C for 12 m, and a final extension at 72°C for 10 min. The PCR product was sequenced with ABIPrism 3730XL Genetic Analyzer (Thermo Fisher Scientific). The RepeatMasker program (https://www.repeatmasker.org/) and dfam database (https://dfam.org/) were employed to identify *Alu* sequences at breakpoint junctions.

### 2.2 RNA analysis

RNA was extracted from PAXgene Blood RNA Kit (PreAnalytiX, Qiagen, Valencia, CA, USA) and used for cDNA synthesis (Superscript III First‐Strand Synthesis SuperMix, Invitrogen, Villebon sur Yvette, France), followed by PCR using a HotstarTaq™ Master Mix Kit (Qiagen, GmbH, Germany). The fragment encompassing exon 9 and 12 of *MLH1* were amplified using the primers: forward: 5′‐CTTACTCTTCATCAACCATCGTCT‐3′ and reverse: 5′‐ CGAGGTCAGACTTGTTGTGGAT‐3′ with the following reaction conditions: 95°C for 15 min followed by 40 cycles of 30 s at 95°C, 30 s at 60°C, 90 s at 72°C and a final 5 min at 72°C. PCR product was then subject to Sanger sequencing.

The SNP c.655A>G was detected in cDNA using the following primers (forward: 5′‐ATTCAGTACACAATGCAGGCATTAG‐3′ and reverse: 5′‐GGGTGCACATTAACATCCACAT‐3′) and in gDNA using the primers (forward: 5′‐ CTCAGCCATGAGACAATAAATCC‐3′ and reverse: 5′‐ GGTTCCAAAATAATGTGATGG‐3′).

### 2.3 Variant interpretation

Variants was interpreted using ACMG criteria‐based (Richards et al., 2015) French NGS‐Diag guidelines (https://anpgm.fr/). As a consensus of the French Cancer Genetics network, relevant tumor phenotype MSI and/or loss of expression was considered as a supporting criterion (PP: Pathogenic Supporting) when displayed in one tumor and could be considered as a strong criterion (PS: Pathogenic Strong) if displayed in tumors from at least two independent families.

## 3 RESULTS

### 3.1 Clinical assessment

**FIGURE 1:**
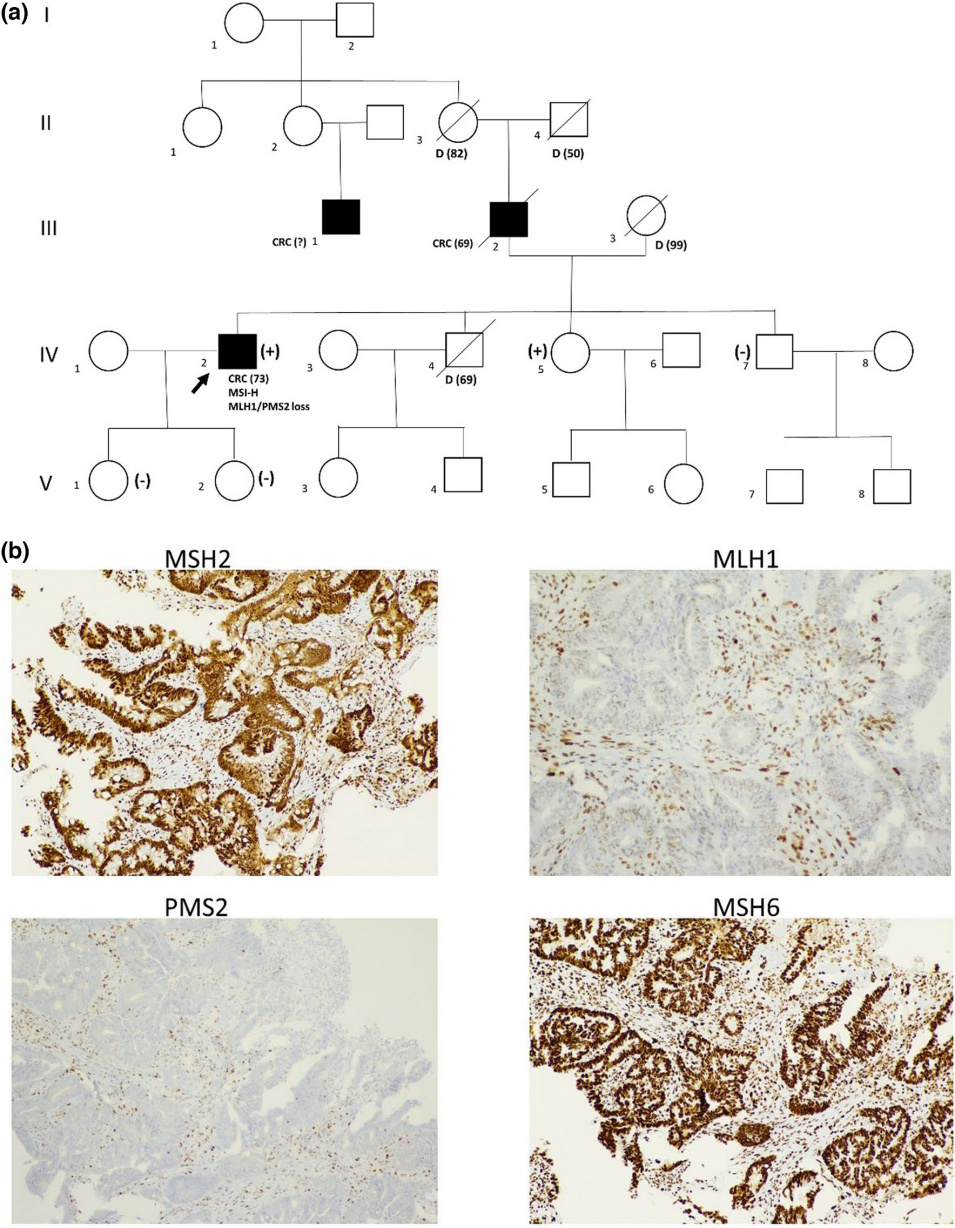
Data from patient. (a) Partial pedigree of the family. Open boxes (male) and circles (female) denote unaffected individuals and filled squares denote individuals with colorectal cancer (CRC) at the age of diagnosis indicated within parentheses. The proband is indicated with an arrow whose tumor showed MSI‐H and loss of MLH1 and PMS2 expression. (+): Subject carrying the *MLH1* variant, (−): Subject not carrying the *MLH1* variant. (b) Immunohistochemistry assay (IHC) of the proband's tumor showing absence of MLH1 and PMS2 staining in tumor cells compared with normal expression in adjacent normal cells, while the MSH2 and MSH6 proteins are normally expressed.

Genetic testing was requested through genetic consulting for a patient who was diagnosed with a right‐sided colon adenocarcinoma (pT3N0) at the age of 73 years. His father developed a colorectal cancer at 69 years old, and a father's cousin developed a colon cancer at an unknown age (Figure 1a). The patient's tumor showed an MSI phenotype and loss of MLH1 and PMS2 expression revealed by immunohistochemistry assay with negative *MLH1* promoter hypermethylation (Figure 1b). Thus, LS was suspected despite of late‐onset cancers in the family and germline variant screening in MMR genes was conducted with NGS strategy after obtaining informed consent.

### 3.2 Genetic analysis

#### 3.2.1 NGS analysis and variant detection

Initial NGS analysis revealed an “insertion” of more than >50 nucleotides of “novel sequences” with a variant frequency of 19% at *MLH1* c.1032_1033. Subsequent sequence blasting analysis showed that this “inserted sequence” was originated from deeper intronic sequence of the intron 11, evidencing that it was a genomic deletion, removing the last six nucleotides together with a large part of intron 11. Because it appeared that only six coding sequences was deleted resulting in a deletion of two amino acids, the biological significance could not be determined. Consequently, it was, at that stage, considered as a variant of unknown significance (VUS).

#### 3.2.2 Variant characterization by LR‐PCR

**FIGURE 2:**
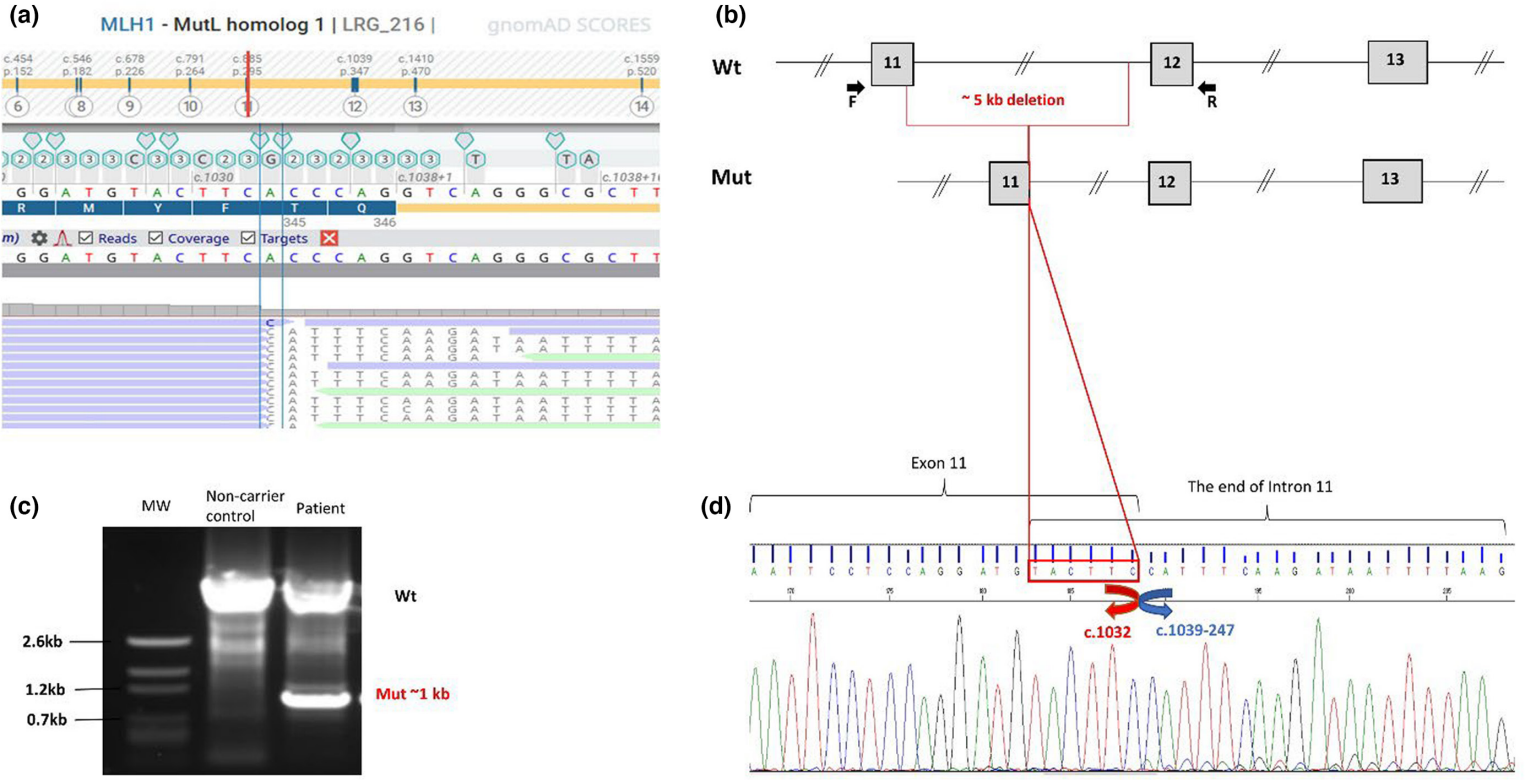
NGS detection and breakpoint characterization of *MLH1* deletion. (a) The two breakpoints of the *MLH1* c.1033_1039‐248 deletion detected by NGS and visualized by Alamut visual plus software. (b) Schematic representation of the *MLH1* normal and mutant alleles, with the deleted region. (c) Long‐range PCR confirmed the presence of the deletion, showing an additional 1 kb PCR product in patient DNA compared to a non‐carrier control. (d) Sanger sequencing of the 1 kb PCR product shows the breakpoint region of the rearrangement, characterized by a small six‐nucleotide element, TACTTC, overlapping the two breakpoints.

We then attempted to better characterize this large deletion, which was of importance for determining the biological consequence of the variant. A long‐range PCR was performed to amplify the region encompassing the exon 11 and the exon 12 including the intron 11, where the breakpoint was located. In the patient's DNA, a smaller additional fragment of about 1 kb was detected in addition to the wild‐type fragment expected to be around 6 kb. Subsequent sequencing enabled to identify the breakpoints which lead to the deletion of a large fragment of approximately 5 kb: c.1033_1039‐248del. The Repeat Masker program did not detect *Alu* sequences or recombination‐associated motifs at upstream and downstream breakpoint junctions. However, we found that a common six‐nucleotide element, TACTTC, was overlapped by two ends of rearranged fragment (Figure 2). Thus, an aberrant recombination through this 6‐base element was very likely the underlying mechanism for this deletion.

#### 3.2.3 RNA analysis

**FIGURE 3:**
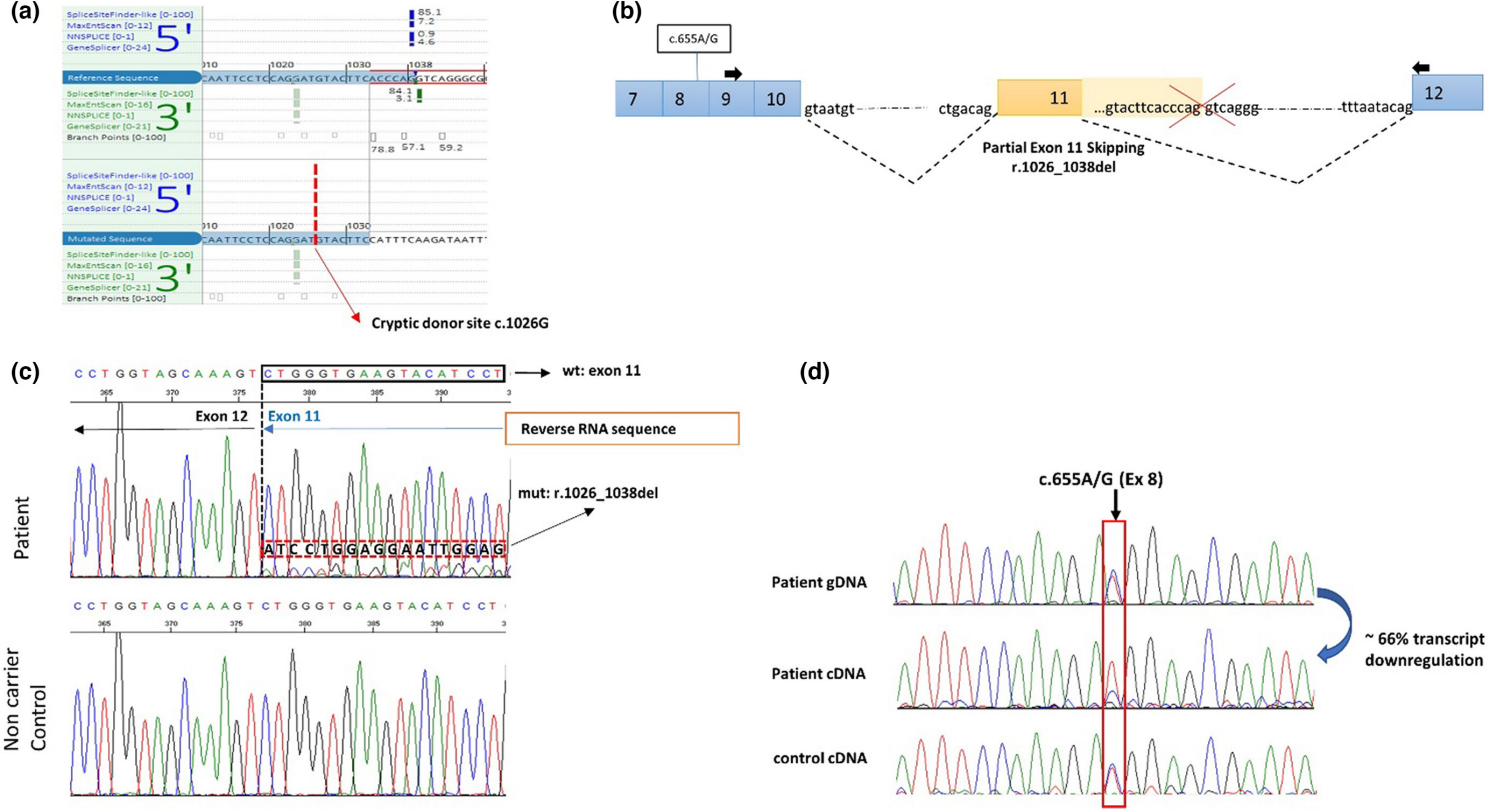
mRNA splicing effect of variant *MLH1* c.1033_1039–248del. (a) In silico prediction by Alamut interface showing changed values for 3′ splice site between normal and altered sequence predicted by four software. (b) Exon involved in an aberrant splicing is colored in orange, and black discontinuous lines represent the splicing patterns. Black arrows represent primers position. (c) Sanger sequencing in patient's cDNA found an overlapped sequence from the end of exon 11, correlating with the sequences of full wild‐type transcript and the aberrant transcript harboring the 13 bp deletion, while only wild‐type transcript was observed in the non‐carrier control cDNA. (d) Sanger electropherogram showing allelic imbalance at polymorphism *MLH1* c.655A>G (reverse sequence) in patient cDNA compared with gDNA and with a control from a non‐carrier cDNA.

Apparently, this genomic deletion caused a deletion of the two amino acids of exon 11 for which the pathogenicity was difficult to define. However, the deletion abolished, at the same time, the splice donor site. To evaluate whether the transcription process was altered, we conducted the transcriptional analysis on the proband's RNA collected with Paxgene tube. Using RT‐PCR, no additional band by gel electrophoresis was observed compared to normal controls. However, Sanger sequencing of the RT‐PCR products revealed a low‐density of aberrant transcript characterized by a deletion of 13 nucleotides: r.1026_1038del. Indeed, this aberrant transcript was caused by the activation of a cryptic 5′ splice donor site at the c.1026_1027 (ATGT), seven nucleotides upstream of the breakpoint within the exon 11. This cryptic splicing led to an out‐of‐frame transcript (r.1026_1038del) resulting in the production a truncating protein. p.Thr345Alafs*13. As the blood sample was not treated with NMD inhibitor puromycin, the low intensity of the aberrant transcript (Figure 3) was very likely caused by the process of nonsense‐mediated mRNA decay (NMD) which degraded mutant mRNA with premature stop codon. This hypothesis was supported by imbalanced allelic expression of the heterozygote SNP c.655A>G at the RNA level (Figure 3), as one of the alleles showing reduced density of expression comparable with that of variant (Figure 3).

Considering these results, we were able to classify this variant as probably pathogenic (Class 4) based on following elements: absence in the general population (PM2), patient's personal and family history of corresponding phenotype (PP), coherent tumors phenotype (PP) and most importantly, functional testing on RNA (PS3).

## 4 DISCUSSION

Recently, NGS technology, coupled with an adapted bioinformatics pipeline, have become a powerful tool to detect structural variants, while some large deletions would probably be missed with conventional PCR‐ or MLPA‐based methods with standard conditions since the mutant allele would not be amplified when primers are in the deleted region.

In the present study, we identified a large deletion of approximately 5 kb occurring in the *MLH1* gene and characterized the breakpoints. This genomic rearrangement was obviously not originated from recombination of *Alu* or other Short interspersed nuclear elements (SINEs) since none of homology was found between such repetitive mobile sequences and deleted sequence including flanking regions. Instead, a 6‐nucleotide sequence (TACTTC) was overlapped by two parts of rearranged fragment, suggesting that a microhomology‐mediated event (MH) involving very short (2–15 bp) homologous sequences was the underlying mechanism. This study highlighted the necessity of using complementary approaches for elucidating complex genomic alterations. More importantly, we showed that functional testing on RNA was crucial to determine the biological consequence of this variant for which the pathogenicity could not be clearly defined from initial experiment. It was sometimes challenging to evaluate the results from RNA splicing assay especially when the mutant allele was weakly represented. The use of an intragenic polymorphism as a control appeared to be useful, demonstrating reduced expression of one heterozygote allele, consistent with the hypothesis of RNA degradation of mutant allele through NMD. In fact, the activity of NMD has well been documented for *MLH1* premature‐termination‐codon (PTC) containing transcripts, evidenced by allelic imbalance ratios between 1.5:1 and 2.2:1 in peripheral blood lymphocytes (Santibanez Koref et al., 2010; Tournier et al., 2004). It is also worthy to note that variants abolishing intron 11 splicing sites seemed to have different transcriptional consequence. In the case described by Nakagawa et al. (2002), they demonstrated that the canonical splicing variant c.1038G>A (AA/GT) caused the inclusion of 59 bp of intron 11 following the activation of an intonic cryptic site. As to our case, as this cryptic site was included in the deleted region, hence another exonic cryptic site 1026_1027GT was activated.

We currently classified this variant as probably pathogenic based on ACMG criteria and derived French consortium criteria. Further studies, such as co‐segregation analysis and confirmation of transcriptional consequence in other carriers of the family or in additional variant‐carrying families, and/or complementary functional testing are required to confirm definitively the pathogenicity of the variant. Certainly, it would be preferable to further demonstrate the causality between this variant and the aberrant transcript with in vitro testing such as mini‐gene experiments. Unfortunately, such tests can hardly be accessible for a clinical diagnosis laboratory. On the other hand, the heterozygous status of the proband remained yet to be ascertained. Indeed, heterozygous status for variant carriers is conventionally established based on a VAF around 50% by NGS. However, the complexity of the variant identified in our patient induced the analytical bias by displaying an altered variant frequency (VAF: 19%). This could be explained by a loss of part of the chimeric reads during the capture and sequence alignment. The possibility of a variant mosaic seemed unlikely considering the familial history. Moreover, the heterozygous status of this variant has been confirmed since the variant was detected in a heterozygous status in his sister (ID: IV‐5). For variant carriers, clinical surveillance should be offered following recommendations including colonoscopy with indigo carmine staining every 2 years starting from the age of 25, gynecological examination with pelvic examination, pelvic ultrasound by external and trans‐vaginal and endometrial biopsies once a year from the age of 30 (Coffin et al., 2019).

In summary, we reported a novel complex *MLH1* variant resulting in aberrant transcription. Our finding suggested that although NGS technologies was sensitive for the variant detection, combined approaches was still required to characterize complex variants and illustrated the importance of functional RNA analysis to determine VUS classification in hereditary cancer syndromes.

## AUTHOR CONTRIBUTIONS

Clementine Legrand is the geneticist who identified the family and consulted the proband. Ahmed Bouras and Qing Wang contributed to the variant identification and interpretation. Cedrick Lefol, Chloé Grand‐Masson, and Eric Ruano performed the experiments. Ahmed Bouras and Qing Wang contributed to writing, editing, and reviewing the manuscript. All authors contributed to the manuscript editing and agreed to the published version of the manuscript.

## FUNDING INFORMATION

There was no specific grant supporting this work.

## CONFLICT OF INTEREST STATEMENT

The authors declare that they have no conflict of interest.

## ETHICAL STANDARDS AND INFORMED CONSENT STATEMENT

Written informed consent was obtained for all patients who were tested and diagnosed within the frame of genetic counseling, in accordance with French law for diagnostic genetic testing. Samples were collected in the frame of care, from patients who consented to a research use of their samples. Testing was done in a hospital laboratory approved for genetic molecular diagnosis. The analyses were performed in accordance with French regulations and the principles of the Declaration of Helsinki.

## Data Availability

The data presented in this study are available on request from the corresponding author. The data are not publicly available due to restrictions of patient privacy.
